# Differentiating human versus non-human bone by exploring the nutrient foramen: implications for forensic anthropology

**DOI:** 10.1007/s00414-017-1662-y

**Published:** 2017-08-21

**Authors:** Vail Johnson, Sophie Beckett, Nicholas Márquez-Grant

**Affiliations:** Cranfield Forensic Institute, Cranfield University, Defence Academy of the United Kingdom, Shrivenham, SN6 8LA UK

**Keywords:** Forensic anthropology, Species identification, Human remains, Nutrient foramen

## Abstract

One of the roles of a forensic anthropologist is to assist medico-legal investigations in the identification of human skeletal remains. In some instances, only small fragments of bone may be present. In this study, a non-destructive novel technique is presented to distinguish between human and non-human long bones. This technique is based on the macroscopic and computed tomography (CT) analysis of nutrient foramina. The nutrient foramen of long bone diaphyses transmits the nutrient artery which provides much of the oxygen and nutrients to the bone. The nutrient foramen and its canal were analysed in six femora and humeri of human, sheep (*Ovies aries*) and pig (*Sus scrofa*) species. The location, position and direction of the nutrient foramina were measured macroscopically. The length of the canal, angle of the canal, circumference and area of the entrance of the foramen were measured from CT images. Macroscopic analysis revealed the femora nutrient foramina are more proximal, whereas humeri foramina are more distal. The human bones and sheep humerus conform to the perceived directionality, but the pig bones and sheep femur do not. Amongst the parameters measured in the CT analysis, the angle of the canal had a discriminatory power. This study shows the potential of this technique to be used independently or complementary to other methods in distinguishing between human and non-human bone in forensic anthropology.

## Introduction

One of the main roles of a forensic anthropologist is to assist medico-legal investigations in the recovery and identification of human skeletal remains [1]. The analysis of bone fragments is often required in mass disasters or fire scenes; in archaeology, the discrimination between human and non-human bone can also be important, for example in the interpretation of funerary practices. It is important that this distinction is made early on in the investigation, so valuable resources, time and money are not wasted.

Macroscopic, microscopic and chemical methods can be employed [2–7]. Comparative anatomy allows discrimination of human and non-human bone by examining gross morphological characteristics [2, 4]. However, in the presence of incomplete, fragmented, damaged or burnt remains, evaluation of the gross morphology is limited [8, 9]. Histological analysis is a method that has been used, but it is destructive and there have been confounding interpretations to the quantitative and qualitative results [3, 9, 10]. Genetic or chemical testing could assist in cases of small, otherwise unidentifiable, fragments [6–11]. Often, however, if the skeletal material is poorly preserved, techniques such as DNA extraction encounter difficulties and may not yield successful results [8, 11]. The use of X-ray diffraction has also been explored to identify species using lattice parameters of cortical bone bioapatite [5, 7]. Normally, multiple methods are to be used together [3], but all these species discrimination studies offer, at times, contradicting results. Further methods to identify the origin of bone fragments are required, particularly those which are non-destructive. This study evaluates a novel approach of using the nutrient foramina, in particular from the analysis of computed tomography (CT) 3D scans to discriminate between human and non-human (human, pig or sheep) and between humeri and femora.

Nutrient foramina transmit the nutrient artery through the diaphysis of the bone, supplying the medullary cavity with 70–80% of the nutrients and oxygen [12, 13]. In long bones, these holes or foramina are found on the diaphysis [14]. A groove, formed by the artery, leads towards the foramen entrance and the nutrient canal travels through the outer cortex of the bone into the medullary cavity [14, 15]. A nutrient vein will sometimes exit the bone through the same foramen [16]. Whilst, it is generally accepted that most long bones are likely to have one dominant nutrient foramen; some long bones may have two or more [13, 17].

The locations of nutrient foramina in bones and their nutrient arteries have been studied for many years [14, 18, 19]. However, the use of the nutrient foramen has not been applied to forensic anthropological cases to assist with species or bone identification. The majority of the research articles published are with reference to dominant human nutrient foramina and their relevance in clinical procedures, such as fracture repair, preventing surgeons from disrupting the vascular supply to osseous tissue during surgery [13, 14, 17]. Studies have focussed on the location, position and number of nutrient foramina in long bones [13–15, 20–22]. Foraminal index [18] is used by researchers to give a measurable indicator of nutrient foramen location, important in surgical procedures [14, 15, 17]. The foraminal index represents the distance of the nutrient foramen from the superior aspect of the bone as a percentage of the total bone length. Some forensic investigations have used the nutrient foramen as an anatomical landmark on the bone for measurements used in sex discrimination in forensic anthropology [23, 24], but have not examined the foramen itself. Research in physical anthropology has also examined arterial, venous and neural foramina in non-metric trait studies, particularly focussing on the presence, absence and number of foramina in the skull [25]. Studies on non-human nutrient foramina have been limited since Hughes’ 1952 publication [18]. Ahn examined nutrient foramen number, location, diameter and nutrient canal directionality as well as the foraminal index in femora and tibiae of German Shepard dogs, with an aim to providing information to orthopaedic veterinary surgeons [26].

Thus, studies have investigated nutrient foramina macroscopically; none, however, have assessed their potential in forensic anthropology as a feature to differentiate between human and non-human species. In addition to adopting similar approaches to other studies, the utilisation of micro-computed tomography in this study has enabled further aspects of nutrient foramina and their nutrient canals to be explored non-destructively.

## Materials and methods

A total of 36 bones were analysed, six femora and six humeri of three species: *Homo sapiens* (human), *Ovis aries* (sheep) and *Sus scrofa* (pig). Three left and three right bones were obtained for each element of each species. The human specimens were loaned from an anatomical collection curated at Cardiff University and all were adult individuals. The sheep and pig bones were obtained fresh from a butcher and were all from skeletally immature individuals. These species were chosen due to their relatively similar size to human remains and their common presence in forensic cases. Each bone was labelled with two letters: ‘H’ for ‘human’, ‘P’ for ‘pig’ or ‘S’ for ‘sheep’, followed by ‘H’ for ‘humerus’ or ‘F’ for ‘femur’. For example, ‘HH’ referred to ‘human humerus’. A number between 1 and 6 was also allocated to differentiate between each bone of a particular skeletal element within each species group.

### Macroscopic analysis

The study focussed on dominant diaphyseal nutrient foramina. These were identified with the 24-gauge hypodermic needle method [13, 14, 21]. If there was more than one dominant foramen present (such as in specimen HF6), the superior foramen was regarded as foramen one and the inferior foramen as foramen two. The anatomical location of the nutrient foramina were recorded: anterior or posterior, medial or lateral. For each nutrient foramen and its canal, directionality was recorded by insertion of the needle into the canal. The needle’s direction was regarded as proximal, distal or perpendicular. Maximum (total) bone length (TL) for all three species was measured using an osteometric board and followed the anthropometric standard measurement specified for human bones [27]. The distance from the superior end of the bone to the nutrient foramina (PE-F) was also measured. For each measurement, three repeats were taken; the mean and standard deviation were calculated. The two measurements were used to calculate the foraminal index for each bone (Eq. 1), following Hughes [18].

1 $$ \mathrm{FI}=\left(\frac{\mathrm{PE}-\mathrm{F}}{\mathrm{TL}}\right)\ \mathrm{x}\ 100 $$

Equation 1, Foraminal index equation. Distance of the nutrient foramen from the superior aspect of the bone, as a percentage of the total bone length. FI is foraminal index, PE-F is the distance of the nutrient foramen from the superior aspect of the bone; TL is the total length of the bone [17].

### CT analysis

Micro-computed tomography data were obtained using a Nikon XTH225 micro CT scanner (tungsten target) and a Varian 2520 flat panel detector. Data were collected using 720 projections and two frames per projection, 500 ms exposure. X-ray settings were 95 kV and 55 μA.

**Fig. 1:**
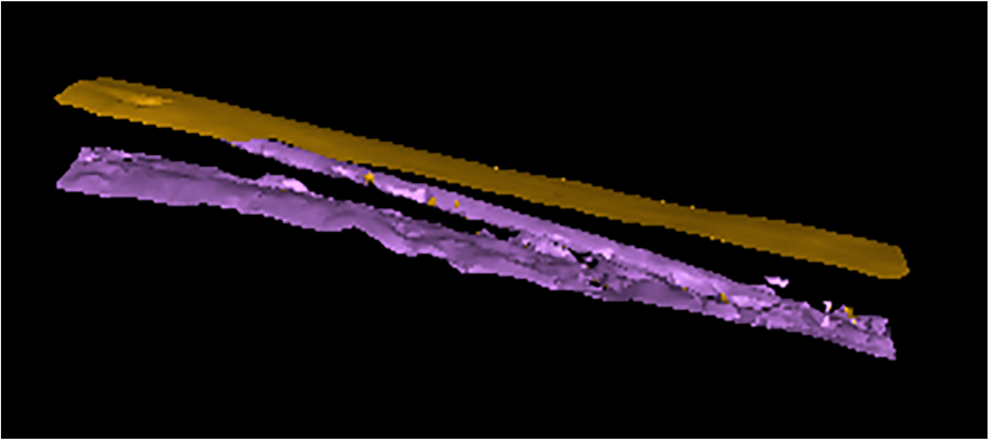
Surface extraction of HH2 nutrient foramen and canal. The upper surface is the exterior of the bone, whilst the lower surface is the interior

**Fig. 2:**
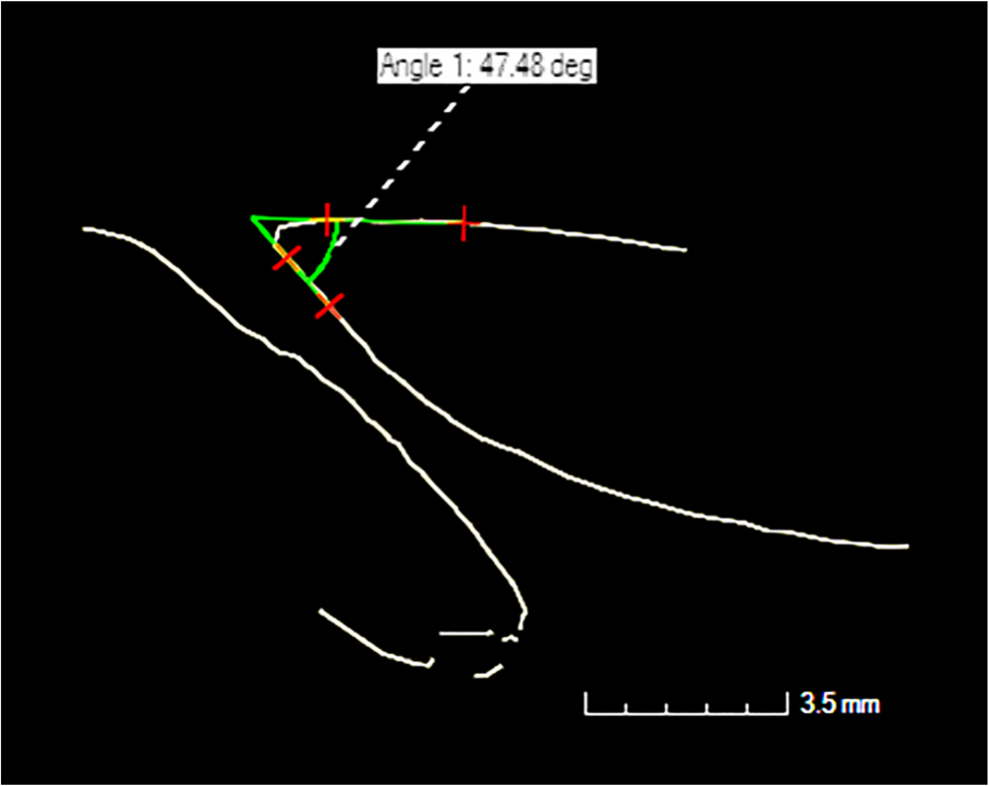
Angle measurement tool for PF4

**Fig. 3:**
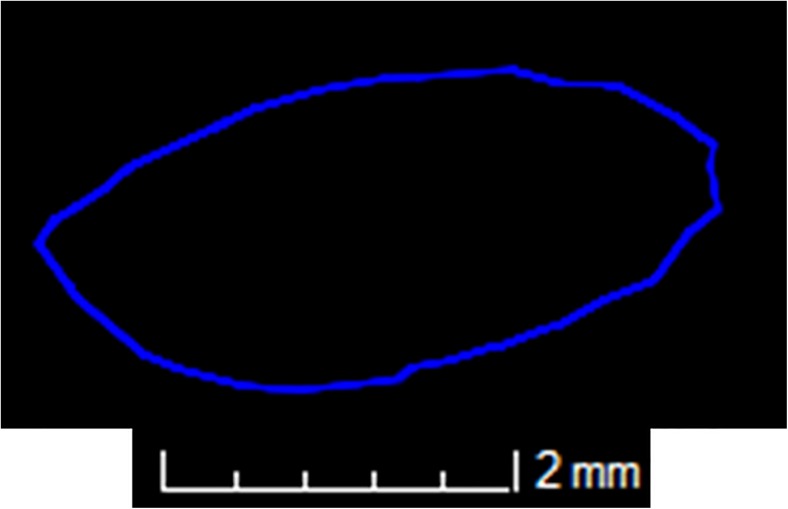
View of nutrient foramen as it enters the bone. This is from a pig femur (PF1) and was classified as elliptical in shape

All scan data were manually reconstructed using CT Pro 3D (Metris) software. Pre-sets for beam hardening and noise reduction levels were used, 3 and 2, respectively. VG Studio Max 2.1 (Volume Graphics) software was employed to visualise the reconstructed data, extract the bone surfaces and the region of interest; to visualise the nutrient foramen and its canal (Fig. 1), and for image orientation within the software scene. The reconstructed image (volume) of the first sample analysed for each set of bones was orientated into position. These were then used as standards to register all other volumes against. Using various planes of view within the software, the following measurements were taken (Figs. 2 and 3):

- Average length of nutrient canal: Measurements of length from each side of the nutrient canal were taken and averaged. Both the cortical bone entrance and the trabecular bone exits were measured, from the start and finish of the previous defined polyline lengths. The distance tool was used in the same plane to measure the distance of the canal.
- Average diameter of nutrient canal: The average represented the hypothetical length in the centre of the nutrient canal. The average was calculated from the two diameters taken at the foramen entrance and exit.
- Angle of nutrient canal (from the outer cortical bone): The angles of the nutrient canal at the cortical bone edge were measured. The angle of the canal from the cortical bone was taken using the angle tool. Figure 2 demonstrates where the angle was taken from the cortical bone to the canal. These measurements were taken from the X-Z slice plane.
- Circumference of the foramen as it enters the bone: The circumferences of the foramina entrances were measured (Fig. 3).
- Area of foramen entrance: The area of the foramen was calculated using the circumference measurement and area of ellipse equation.

### Statistical analysis

Mean and standard deviation values were calculated for each measurement taken. Statistical analysis was performed using Minitab software. Both a one-way and a two-way analysis of variance (ANOVA) were performed on the CT measurement data (mean length, diameter, angle, circumference and area of foramen).

## Results

### Macroscopic analysis

Consistent differences were observed for both anatomical location and directionality, with respect to species and bone (Table 1). All human femoral nutrient foramina were found on the posterior surface (alongside the *linea aspera*, a site for muscle attachments running along the shaft), whereas all sheep and pig femoral foramina were located on the anterior bone surface. All human femoral nutrient foramina were also found to have a proximal directionality, different to all sheep and pig femoral foramina which had a distal directionality. A human versus non-human difference was also observed for anatomical location on all humeral bones. Sheep and pig humeral foramina were found on the posterior and all human humeral nutrient foramina were found on the medial surface (next to the medial supracondylar ridge). This was not the case for humeral foramina directionality. All pig humeral nutrient foramina were distinctive due to their transverse directionality as opposed to the distal directionality observed for all human and sheep humeral nutrient foramina.

**Table 1:** Anatomical location, directionality and foraminal index results, summarised by species group and bone element

*n* = 6 per group	Anatomical location	Directionality	Mean foraminal index (%)	Foraminal index range (%)
Human femur	Posterior	Proximal	38	35–43
Pig femur	Anterior	Distal	27	25–29
Sheep femur	Anterior	Distal	26	21–25
Human humerus	Medial	Distal	59	56–63
Pig humerus	Posterior	Transverse	59	58–61
Sheep humerus	Posterior	Distal	56	49–61

Proportionally, the human femoral nutrient foramina were located closer to the mid-point of the total bone length. The mean foraminal index calculated for human femoral bones was approximately 10% greater than those obtained for pig and sheep femoral bones (Table 1). This difference was statistically significant (*p* < 0.05) and there was no significant difference between the sheep and pig femoral values. There were no significant differences between species with respect to humeral nutrient foramina values for mean foraminal index (Table 1). However, contrary to the proportional location of femoral foramina; in general, humeral foramina were located distal to the mid-point of the total bone length. All humeral mean foraminal index values were greater than 50%, with the exception of one sheep humerus with a calculated value of 49%.

Only one bone, a human femur (HF6), was found to have two dominant nutrient foramina. Previous studies have suggested separating the results for bones with one nutrient foramen and those with multiple nutrient foramina [18]. The superior foramen was included in the results shown in Table 1. Whilst, the foraminal index for the more inferiorly located foramen was inevitably greater than the mean femoral value presented in Table 1, both location and directionality were consistent with all other dominant human femoral foramina.

### CT analysis

The mean values obtained for nutrient foramen length, diameter, angle, circumference and entrance area (as defined in the methods section) are presented by species and bone element in Table 2. For both length and diameter measurements, all values within a group were within one standard deviation of the mean. Although there was overlap of values between groups, both human femoral and humeral nutrient canal mean length values were significantly greater than the respective mean values obtained for pig and sheep (*p* < 0.01). There was no significant difference between human bone elements with respect to mean length values. In general, mean values obtained for human femoral and humeral diameter measurements were greater than the respective measurements obtained for pig and sheep, with the exception of pig femoral diameters for which the largest mean value was obtained and a significant difference was observed (*p* < 0.01). Similar to length measurement results, there was no significant difference between human bone elements with respect to mean diameter values. The mean values obtained for sheep femoral and humeral circumference measurements were significantly different from the respective values obtained for human and pig bone elements (*p* < 0.01) but there were no significant differences between human and pig groups with respect to circumference. In general, foramina entrance shapes were elliptical (see Fig. 3). The largest mean values for both femora and humeri were obtained for pig bones, in terms of area of entrance. However, there were no significant differences observed between species or bone groups with respect to this measurement (*p* > 0.05). Consistent with a transverse directionality, the largest mean value for the angle measurement was obtained for pig humeri and this value was significantly different from all other groups (*p* < 0.01). The angles of the nutrient canals in human bones were more acute compared to the other species, especially in terms of the humerus, for which a significant difference was observed (*p* < 0.01).

**Table 2:** Mean values for measurements obtained from computed tomography image data, summarised by species group and bone element. Standard deviations for mean values are presented in parentheses

*n* = 6 per group	Length (mm)	Diameter (mm)	Circumference (mm)	Area of entrance (mm^2^)	Angle (°)
Human femur	28.4 (16.7)	3.9 (2.2)	8.2 (2.2)	3.2 (1.1)	20.2 (7.9)
Pig femur	14.7 (1.5)	5.7 (0.9)	8.5 (1.8)	5.0 (1.9)	35.3 (12.4)
Sheep femur	5.0 (1.2)	2.6 (0.5)	7.3 (0.9)	3.3 (0.7)	63.2 (18.9)
Human humerus	37.2 (7.6)	3.7 (1.5)	8.8 (3.4)	2.8 (1.4)	7.2 (3.0)
Pig humerus	8.2 (0.6)	2.7 (0.3)	6.8 (0.8)	3.3 (0.7)	98.5 (12.5)
Sheep humerus	12.1 (0.8)	2.2 (0.3)	4.3 (1.5)	1.3 (0.8)	37.7 (6.3)

As with Table 1, only results for the superior foramen were included in Table 2, in the case of the human femur with two dominant foramina.

## Discussion

### Macroscopic analysis

This study has shown that human nutrient foramina are consistently located on specific surfaces, close to anatomical landmarks and this finding is consistent with those of others in previous studies [13, 14]. Nutrient foramina of other species are also consistently located on specific surfaces but away from anatomical landmarks. Previous studies have referred to a directionality rule with regards to nutrient foramen differences between bone elements [18, 26]. The rule specifies that nutrient foramina and their nutrient canals in upper limb long bones travel distally (towards the elbow joint); whereas the lower limb nutrient foramina directionality is proximal (away from the knee joint) [18]. The study presented here, found that human bones comply with this rule whereas pig bones do not. In the case of sheep long bones, the upper limb complied with the rule but the lower limb did not. This finding corroborates those of other researchers who found that directionality did not agree with the rule for a number of non-specified tetrapods [18]. More specifically, Ahn found also this disagreement to the rule in a number of dog species [26]. The consistent differences between species and bone element in terms of anatomical location and directionality would enable differentiation using these two features. In practice, it might be difficult to determine anatomical location and directionality for small fragments; however, the observation of these features in conjunction with specific landmarks such as the *linea aspera* and the medial supracondylar ridge in the femur and humerus, respectively, would enable the confirmation of bone of human origin. Further research might enable pig humeri to be distinguished based on recognition of transverse directionality alone and in cases where a small bone fragment possessing a nutrient foramen had been identified as human by other means (such as DNA analysis), directionality would enable classification by bone element. The foraminal index would not be particularly useful in determining species or bone element in forensic contexts that involve fragmented bone, as the entire bone is required for its calculation. The whole-bone observations used in this study cannot be utilised for fragmentary remains. However, they enabled as full a macroscopic analysis as possible and have certainly contributed to enhancing knowledge of nutrient foramen generally.

### CT analysis

The results presented in this study clearly demonstrate that there are significant differences between species and bone elements in terms of quantitative nutrient foramen parameters, measured using computed tomography data. Based on the results of this study, measurement of a nutrient canal length greater than 20 mm would enable the identification of a fragment as human, even if bone element could not be distinguished. Applied in isolation, the diameter, circumference and area of entrance measurements would not be useful discriminators for either species or bone element due to a lack of statistical significant differences. However, measurement of an angle less than 20° would enable identification of human origin and an angle less than 10° would enable identification of a humeral, as opposed to a femoral, fragment, based on the results of this study. Furthermore, as the angle measurement is related to directionality, measurement of an angle greater than 80 ° would be indicative of bone of porcine origin. The discriminating power of canal length and angle measurements could potentially be increased through use of both parameters in a combined assessment.

CT analysis is becoming more routinely used in forensic cases, especially for fragmented remains [28–30], and in many cases, several fragments can be scanned simultaneously. It is a non-destructive technique that can be used in conjunction with other methods and also to triage bone fragments ahead of destructive and expensive techniques such as DNA analysis. The results of this study may therefore prove to be a valuable tool for forensic anthropology in the future.

## Conclusion

This paper is the first study of its kind and has identified a novel approach for the use of nutrient foramina in forensic anthropology. The authors acknowledge that further research is necessary to consolidate the results presented in this preliminary study. Due to specimen availability and time constraints, it was not possible to mitigate for several factors such as sample size and comparison of all adult remains. Therefore, future studies should consider; a wider range of species, larger sample sizes, both adult and juvenile remains of each species, fresh and dry bone, a range of foramina types such as secondary nutrient foramina or metaphyseal foramina and, pathological and diagenetic effects. Parameters with large standard deviations of the mean for repeat measurements such as angle should be reviewed with the aim of improving repeatability within the measurement acquisition method. However, it has been clearly demonstrated here that the nutrient foramen can be used to discriminate between human and non-human bone. It is proposed that differences between, for example, fresh and dry bone will have negligible effects on the parameters measured and that the results of this study will be valid with respect to further work. Indeed, this practical and non-destructive method has considerable potential and value which should assist in forensic cases where there are fragmentary skeletal remains. Any further work would also contribute to knowledge of human nutrient foramina for medical applications.
